# Adherence to Antiplatelet Medications among Persistent and Non-Persistent Older Patients with Peripheral Arterial Disease

**DOI:** 10.3390/biomedicines9121800

**Published:** 2021-11-30

**Authors:** Martin Wawruch, Jan Murin, Tomas Tesar, Martina Paduchova, Miriam Petrova, Denisa Celovska, Petra Matalova, Beata Havelkova, Michal Trnka, Emma Aarnio

**Affiliations:** 1 Institute of Pharmacology and Clinical Pharmacology, Faculty of Medicine, Comenius University, 811 08 Bratislava, Slovakia; miriampetrova1@gmail.com; 2 1st Department of Internal Medicine, Faculty of Medicine, Comenius University, 813 69 Bratislava, Slovakia; jan.murin@gmail.com (J.M.); denisa.celovska@gmail.com (D.C.); 3 Department of Organisation and Management of Pharmacy, Faculty of Pharmacy, Comenius University, 832 32 Bratislava, Slovakia; 4 Department of Angiology, Health Centre, 917 01 Trnava, Slovakia; martina.paduchova@medena.sk; 5 Department of Pharmacology, Faculty of Medicine and Dentistry, Palacky University, 775 15 Olomouc, Czech Republic; petra.matalova@fnol.cz; 6 General Health Insurance Company, 851 04 Bratislava, Slovakia; beata.havelkova@vszp.sk; 7 Institute of Medical Physics, Biophysics, Informatics and Telemedicine, Faculty of Medicine, Comenius University, 813 72 Bratislava, Slovakia; michal.trnka@fmed.uniba.sk; 8 Institute of Biomedicine, University of Turku, 20014 Turku, Finland; emma.aarnio@uef.fi; 9 School of Pharmacy, University of Eastern Finland, 70211 Kuopio, Finland

**Keywords:** peripheral arterial disease, adherence, antiplatelet, persistence, co-payment, general practitioner, new user

## Abstract

Secondary prevention of peripheral arterial disease (PAD) includes administration of antiplatelet agents, and adherence to medication is a requirement for an effective treatment. The aim of this study was to analyse adherence measured using the proportion of days covered (PDC) index separately in persistent and non-persistent patients, and to identify patient- and medication-related characteristics associated with non-adherence in these patient groups. The study cohort of 9178 patients aged ≥ 65 years in whom PAD was diagnosed in 1/–12/2012 included 6146 persistent and 3032 non-persistent patients. Non-adherence was identified as PDC < 80%. Characteristics associated with non-adherence were determined using the binary logistic regression model. In the group of persistent patients, 15.3% of subjects were identified as non-adherent, while among non-persistent patients, 26.9% of subjects were non-adherent to antiplatelet medication. Administration of dual antiplatelet therapy (aspirin and clopidogrel) and a general practitioner as index prescriber were associated with adherence in both patient groups. Our study revealed a relatively high proportion of adherent patients not only in the group of persistent patients but also in the group of non-persistent patients before discontinuation. These results indicate that most non-persistent PAD patients discontinue antiplatelet treatment rapidly after a certain period of adherence.

## 1. Introduction

Peripheral arterial disease (PAD) represents a flow-limiting condition caused by narrowing of the peripheral arteries mostly due to atherosclerosis [1]. In our manuscript, PAD refers to atherosclerotic disease in the arteries of lower limbs. According to the systematic review and analysis of Fowkes et al. [2], 202 million people globally were living with PAD in 2010 and during the preceding decade, the number of subjects with PAD increased by 28.7% in low or middle-income countries and by 13.1% in high-income countries. PAD incidence and prevalence are sharply age–related, rising >10% among patients in their 60s and 70s [3]. Clinical manifestations of PAD include asymptomatic patients, those with intermittent claudication, and patients with critical limb ischemia or acute limb ischemia. PAD patients have an increased risk of coronary artery disease mortality, cardiovascular (CV) mortality and all-cause mortality [1,2,3,4,5,6]. Risk factors of PAD include common risk factors of atherosclerosis, e.g., smoking, hypertension, hypercholesterolemia, diabetes mellitus and chronic kidney disease. In addition to treatment of modifiable risk factors, treatment of PAD includes administration of secondary preventive medication–antiplatelet agents, statins and inhibitors of angiotensin converting enzyme/angiotensin receptor blockers [7,8,9].

Adherence to medications represents the basic precondition of treatment of PAD. Adherence includes three interrelated phases: initiation, implementation and persistence [10,11]. In the literature, there are almost no studies focused on the analysis of adherence to antiplatelet medications in PAD patients. Qvist et al. [12] analysed adherence to antiplatelet medications and statins among 65–74 year old men diagnosed with abdominal aortic aneurysm or PAD. Proportion of days covered (PDC) ≥ 80% was used as a threshold of adherence. Among non-users at baseline, 60% were adherent to antiplatelet treatment, while among users at baseline, 89% were adherent. We recently carried out a study focused on non–persistence with antiplatelet agents in older PAD patients [13]. In the group of 9178 patients aged ≥ 65 years, non-persistence was identified at the end of the 5-year follow-up period in 3032 (33.0%) subjects. Patient and medication-related characteristics associated with non-persistence were determined.

There are various methods to analyse the implementation phase of adherence, and adherence measurement using indexes such as PDC can be applied to analyse register-based data [14]. Our study was aimed at (a) the analysis of adherence measured using the PDC index separately in the groups of persistent and non-persistent patients; (b) analysis of adherence among non-persistent patients depending on when becoming non-persistent during the 5-year follow-up; and (c) identification of patient- and medication-related characteristics associated with non-adherence separately in persistent and non-persistent patients. To the best of our knowledge, in the literature there is no similar study evaluating these issues specifically in older (aged ≥ 65 years) PAD patients.

## 2. Materials and Methods

### 2.1. Database and Study Population

The study group of our register-based retrospective cohort study included 9178 antiplatelet agent users aged ≥ 65 years in whom PAD was diagnosed between 1 January and 31 December 2012. This sample was analysed in our previous manuscript [13] in which the derivation of the study cohort is described in detail. At the end of the 5-year follow-up period, 3032 non-persistent subjects were identified in this cohort based on the presence of a treatment gap period of ≥ 6 months without antiplatelet medication prescription. The General Health Insurance Company, which is the largest health insurance provider in the Slovak Republic, represented the source of data for our study.

### 2.2. Analysis of Adherence

Adherence was analysed using the PDC index. PDC was calculated as the number of days covered with tablets of antiplatelet agents divided by the number of days the patient was persistent (i.e., in persistent patients, the number of days of their whole follow-up and in non-persistent patients, the number of days before discontinuation). In the case of non-persistent patients, only their persistent period was considered for calculation of PDC since the focus of the study was on the implementation phase of adherence. Using their whole follow-up (persistent and non-persistent period) as a denominator of PDC would have led to overestimation of their non-adherence. We also wanted to study whether the factors associated with non-adherence differed between persistent and non-persistent patients and, therefore, analyses were performed separately in these patient groups. The once daily dosing of antiplatelet medications (aspirin, clopidogrel) was considered for analyses. The only exception was ticlopidine which is administered twice a day. PDC threshold < 80% was used to define non-adherence [14].

In the group of non-persistent patients, an additional analysis of adherence depending on the length of persistence during the 5-year follow-up divided into periods of 12 months was performed. The mean PDC values were calculated for each 12-month period.

### 2.3. Factors Associated with Non-Adherence

The same patient- and medication-related characteristics that were analysed in our previous study [13] as factors potentially associated with non-persistence were used in this study to identify factors associated with non-adherence. The data on particular characteristics were collected at the time of inclusion into the study except for the history of CV events where a look-back period of 5 years was used.

Analyses of factors associated with non-adherence were performed separately in the groups of persistent and non-persistent patients. Additionally, this analysis was performed in the whole study group, which included persistent and non-persistent patients.

### 2.4. Statistical Analysis

Continuous variables were characterised as means ± standard deviations and categorical variables as frequencies and percentages.

To compare categorical variables between two groups, the χ^2^-test was applied. To analyse the differences in continuous variables between two groups, the Mann–Whitney U test was used. In the case of more than two groups, continuous variables were compared using the Kruskal–Wallis test. These two non-parametric tests were applied because of the non-Gaussian distribution of analysed continuous variables. The normality of distribution was tested using the Kolmogorov–Smirnov test.

To identify the association between patient- and medication-related characteristics and non-adherence, the binary logistic regression model was used. Odds ratios and corresponding 95% confidence intervals were calculated for each factor [15].

All statistical tests were performed at the significance level of α = 0.05. The statistical software IBM SPSS for Windows, version 27 (IBM SPSS Inc., Armonk, NY, USA), was applied.

### 2.5. Sensitivity Analyses

Analyses of factors associated with non-adherence using different PDC thresholds (50%, 60%, 70% and 90%) than the threshold used in the main analysis (80%) were performed. A logistic regression model with a shorter 3-year follow-up period was created in order to assess the effect of a different length of the follow-up period than that used in the main analysis (5-year follow-up).

## 3. Results

The baseline characteristics of adherent and non-adherent patients are presented in Table 1. In the group of 6146 persistent patients, 942 (15.3%) subjects were identified as non-adherent (PDC < 80%), while among 3032 non-persistent patients, 817 (26.9%) were found to be non-adherent to antiplatelet medication (*p* < 0.001 according to the χ^2^-test). The mean PDC in the whole study population was 89.6 ± 13.5%. In the group of persistent patients, the mean value of PDC was found to be higher (90.7 ± 12.1%) than in the group of non-persistent patients (87.4 ± 15.7%) (*p* = 0.007 according to the Mann–Whitney U test).

In the analysis of adherence within different 12-month periods during which non-persistent patients discontinued antiplatelet treatment, significantly higher proportions of adherent than non-adherent patients were found within all five 12-month periods (*p* < 0.001 according to the χ^2^-test) (Table 2). The highest proportion of non-adherent patients (44.2%) was found in the period between 13 and 24 months. The mean values of PDC significantly differed among particular 12-month periods (*p* < 0.001 according to the Kruskal–Wallis test). However, in each period the mean PDC value was over 80%.

In the group of persistent patients, university education, dual antiplatelet therapy (DAPT–aspirin + clopidogrel), a higher co-payment, a general practitioner being the index prescriber and administration of statins were associated with adherence (Table 3). In the group of non-persistent patients, history of ischemic stroke, administration of clopidogrel or DAPT, being a new antiplatelet agent user, a general practitioner being the index prescriber and administration of cardiac glycosides were associated with adherence. Similar characteristics were found in the analysis of the whole study group, which included persistent and non-persistent patients (Supplementary Table S1). The only exception was bronchial asthma/chronic obstructive pulmonary disease (COPD), which represented the only factor associated with increased probability of non-adherence in this study.

### Sensitivity Analyses

In the case of sensitivity analysis using different PDC thresholds to define non-adherence (50%, 60%, 70% and 90%), the following percentages of non-adherent patients were found in persistent and non-persistent patients: 0.8% and 1.9%; 3.5% and 7.6%; 7.9% and 16.6%; 32.0% and 42.0%, respectively. In the logistic regression models using these PDC thresholds, similar factors associated with adherence were found as those identified in the main model, which used the 80% PDC threshold (Supplementary Table S2). Additionally, epilepsy was associated with non-adherence among non-persistent patients in models with 50% and 60% PDC thresholds and a higher number of medications was associated with non-adherence among non-persistent patients in the model with 90% PDC threshold.

In the case of a shorter 3-year follow-up period, 1125 (17.5%) from persistent patients (*n* = 6419) were identified as non-adherent, and 722 (26.2%) from non-persistent patients (*n* = 2759) were non-adherent. The mean PDC in the whole study sample was 89.3 ± 13.8%. In the group of persistent patients, the mean PDC was 89.9 ± 12.7%, and in the group of non-persistent patients it was 87.9 ± 16.0% (*p* = 0.002 according to the Mann–Whitney U test). In the model with a 3-year follow-up period, similar factors associated with adherence were found as those identified in the main analysis with a 5-year follow-up (Supplementary Table S3). The only differences were that university education in persistent patients and a general practitioner being index prescriber and administration of cardiac glycosides in non-persistent patients were not associated with adherence in the model with a shorter 3-year follow-up period.

## 4. Discussion

Our study revealed a significantly higher proportion of non-adherent patients in the group of non-persistent patients (26.9%) in comparison with the group of persistent patients (15.3%). Consequently, the mean value of PDC was higher in the group of persistent patients compared to non-persistent ones. Based on these results, certain level of non-adherence before discontinuation in non-persistent patients could be expected. However, in both groups, the mean PDC values exceeded the 80% adherence threshold, and over 70% of non-persistent patients were adherent before discontinuation.

Our results on the prevalence of adherence are within the range of estimates presented in previous studies. As mentioned above in the Introduction, in the study by Qvist et al. [12], among non-users of antiplatelet medication at baseline 60% were adherent (PDC ≥ 80%), while among users at baseline 89% were adherent. The study focused on the analysis of adherence to antiplatelet and statin therapy in men aged 65–74 years with abdominal aortic aneurysm or PAD. Kuepper-Nybelen et al. [16] reported only 21.8% patients to be adherent to antiplatelet drugs determined by PDC ≥ 80%. Their prospective cohort study based on claims data analysed the association of long-term adherence to evidence-based drugs after acute MI with all-cause mortality. Sanfélix-Gimeno et al. [17] analysed adherence to evidence-based therapy after acute coronary syndrome in a retrospective population-based cohort study. Adherence was defined using a 75% threshold of PDC. According to this threshold, 69.9% of patients taking antiplatelet medications were adherent.

To analyse adherence in more detail among non-persistent patients, the proportions of adherent and non-adherent patients who discontinued antiplatelet therapy during particular 12-month period of the 5-year follow-up period were analysed. Surprisingly, adherent patients prevailed over non-adherent ones in all periods. This indicates that most non-persistent patients were adherent until a certain point of time when they rapidly discontinued antiplatelet treatment rather than being non-adherent and implementing their medication regimen poorly before discontinuation. This result suggests that PAD patients are not sufficiently aware of the necessity of life-long administration of antiplatelet treatment. This may be associated with the fact that the use of antiplatelet medication in the treatment of PAD represents a prevention of the progress of the disease and CV events [4,5,6,7]. Patients do not perceive direct benefit of treatment and they consider it to be unnecessary after a certain period of time during which they adequately adhered to antiplatelet medication.

In our study, university education was associated with adherence in the group of persistent patients. This result may indicate better awareness of the importance of antiplatelet treatment in PAD patients with higher education level. This awareness is particularly important in the case of secondary preventive medications when the beneficial effect requires long-term use. Similar to our results, in the study by Uchmanowicz et al. [18], patients with a higher education tended to be more adherent to antihypertensive medication. Their cross-sectional study was aimed at identifying demographic, socioeconomic and clinical factors that affect adherence in older adults with hypertension. In the systematic review by Czarny et al. [19], lower educational level was associated with non-adherence. This review was focused on adherence to DAPT in patients after coronary stenting. A higher educational background was associated with a higher percentage of patients fully adherent to medication in the study by Cordero et al. [20]. This study was focused on the analysis of adherence to medications used in the secondary prevention of CV events in Spain. They measured adherence with the Morisky–Green adherence questionnaire [21]. In addition, a lower level of education was associated with a decreased adherence also according to the systematic review and meta-analysis of factors influencing medication adherence among pre-dialysis chronic kidney patients by Seng et al. [22]. Antihypertensive agents represented the most studied medication class.

History of ischemic stroke appeared as a factor associated with adherence to antiplatelet medication in non-persistent patients in our study. History of ischemic stroke was associated with persistence in our previous study [13]. These results suggest that patients with a history of ischemic stroke tend to persist with antiplatelet treatment and if they discontinue, they had adhered adequately before discontinuation. These findings indicate an increased patient awareness of the importance of antiplatelet medication in the case of other concomitant conditions, such as ischemic stroke, whose treatment also requires administration of antiplatelet agents [23,24].

Administration of clopidogrel was associated with adherence in the group of non-persistent patients, while administration of DAPT (aspirin and clopidogrel) was associated with adherence in both groups of persistent and non-persistent patients. Administration of clopidogrel or DAPT was associated with persistence in our previous study [13]. DAPT is administered in patients who underwent percutaneous coronary intervention after MI. These results indicate again a better patient awareness of the necessity of antiplatelet medication in the treatment of concomitant diseases whose secondary prevention also requires administration of antiplatelet medication (e.g., MI) [25]. Using more than one antiplatelet medication has been previously shown to be associated with better adherence also to other medications, as patients with a combination of antiplatelet agents were more likely to be adherent to antihypertensive medication in the study by Yue et al. [26].

Being a new antiplatelet medication user, i.e., a patient in whom antiplatelet medication administration was initiated in association with PAD diagnosis, was associated with adherence in the group of non-persistent patients. On the other hand, in our previous study [13], being a new antiplatelet medication user was associated with non-persistence. These results may indicate that new users tend to discontinue the treatment, possibly because of problems with tolerance of medication at the beginning of therapy (e.g., adverse effects). However, before discontinuation they have had good adherence compared to old users who seem to have had worse adherence before discontinuing the treatment. On the other hand, being a new user of lipid-lowering medications was associated with non-adherence according to the systematic review by Lopes and Santos [27] focused on determinants of non-adherence to medications used in the treatment of dyslipidaemia. Being a new statin user represented a factor associated with increased non-adherence also in the systematic review and meta-analysis by Ofori-Asenso et al. [28].

Patient´s co-payment represented a factor associated with adherence in the group of persistent patients. Patient´s co-payment was associated with persistence in our previous study [13]. Prescription drug cost-sharing in the form of patient´s co-payment is generally considered a potential barrier to optimal adherence [29]. According to the systematic review by Goldman et al. [30], increased cost sharing is associated with worse adherence, and more frequent discontinuation of medications. Higher co-payments have been reported to be associated with an increased statin non-adherence in the systematic review and meta-analysis by Ofori-Asenso et al. [28]. The design of our study does not make it possible to explain why higher patient´s co-payment is associated with adherence. One possible explanation may be the relatively low co-payment (on average of 1.4 EUR per month) which does not represent a significant financial burden even for older patients.

The prescription of antiplatelet medication by general practitioner at the time of PAD diagnosis (index prescriber) was associated with adherence in both groups of persistent and non-persistent patients. General practitioner as index prescriber represented a factor associated with persistence with antiplatelet medication in our previous study [13]. These results indicate the key role of general practitioners in achieving patients´ adherence to this secondary preventive medication. As mentioned above, in the case of antiplatelet medication, adherence depends on patients´ awareness of the importance of this treatment in secondary prevention of PAD. For this reason, the general practitioner´s explanation of this matter has a significant impact on adherence. Gale et al. [31] concluded in their qualitative study focused on patient and general practitioner attitudes to taking medication to prevent CV disease that general practitioners must take care to explain what preventive medication is and why it is recommended.

Administration of cardiac glycosides represented a characteristic associated with adherence in the group of non-persistent patients, while administration of statins was associated with adherence in the group of persistent patients in our study. Statins also represent secondary preventive medication in treatment of PAD similarly to antiplatelet agents [4]. This result may suggest that patients who are aware of the importance of secondary prevention of PAD adhere to both statins and antiplatelet agents.

In the logistic regression models performed separately in the groups of persistent and non-persistent patients, only factors associated with adherence were found. None of the evaluated characteristics was associated with non-adherence. In the logistic regression model, which included both persistent and non-persistent patients, similar factors were associated with adherence as those identified in the separate models. One exception was bronchial asthma/chronic obstructive pulmonary disease (COPD) which represented the only factor associated with non-adherence found in our study. Ofori-Asenso et al. [28] reported in their systematic review and meta-analysis respiratory disorders (COPD or asthma) as being associated with increased statin non-adherence. Similarly, COPD represented a factor associated with non-adherence in the systematic reviews by Czarny et al. [19] and Lopes and Santos [27].

Our study has some limitations which should be considered when interpreting the study results. The database of the General Health Insurance Company was originally created for health insurance and not research purposes. It does not make it possible to distinguish who was responsible for medication discontinuation, whether it was the physician or the patient. It is also impossible to determine whether patients took their medications as prescribed by the physician as it cannot be established whether medications were truly taken by patients. The database of the General Health Insurance Company does not include information on the grade and severity of PAD, and consequently it was impossible to assess these clinical variables. In addition to antiplatelet medication, statins and inhibitors of angiotensin-converting enzyme/angiotensin receptor blockers are also used in the secondary prevention of PAD [4,7]. We analysed adherence to statin treatment in our recent manuscript [32]. However, the study presented in this manuscript is focused solely on antiplatelet agents. On the other hand, the large sample size and the detailed information on patients’ comorbid conditions and medications represent the strengths of our study.

## 5. Conclusions

Our study revealed a relatively high proportion of patients with adherence not only in the group of persistent patients but also in the group of non-persistent patients before discontinuation. These results indicate that non-persistent PAD patients discontinue antiplatelet treatment rapidly after a certain period of adherence, which may suggest an insufficient awareness of the importance of life-long administration of this medication. Except for bronchial asthma/COPD, only factors associated with adherence were identified in our study. Patients without these protective characteristics are more likely to be non-adherent. In these patients, special attention should be paid to educating them about the importance of antiplatelet medication in secondary prevention of PAD. Since to achieve the beneficial effects of secondary prevention of PAD, regular long-term administration of medication is required, non-adherence represents a serious public health issue. To address this issue, health care professionals should pay attention to patients’ implementation of the medication regimen to ensure the benefits of medications. Medication adherence should be monitored by health care professionals in order to identify problem patients. As our results show, patients need motivating also during implementation of the medication regimen and not only at the time of medication initiation.

## Figures and Tables

**Table 1:** Baseline characteristics of the study cohort (*n* = 9178).

Factor	Persistent Patients (*n* = 6146)			Non-Persistent Patients (*n* = 3032)		
	Adherent (*n* = 5204)	Non-Adherent (*n* = 942)	*p*	Adherent (*n* = 2215)	Non-Adherent (*n* = 817)	*p*
*Socio-demographic characteristics*						
Age	75.8 ± 7.1	76.9 ± 7.1	<0.001 *	73.7 ± 6.1	73.6 ± 5.9	0.697 *
Female sex	2901 (55.7)	512 (54.4)	0.428	1338 (60.4)	534 (65.4)	**0.013**
University education	350 (6.7)	43 (4.6)	**0.013**	183 (8.3)	61 (7.5)	0.475
Employed patients	222 (4.3)	36 (3.8)	0.532	144 (6.5)	46 (5.6)	0.380
*History of cardiovascular events* ^a^						
History of ischemic stroke	1134 (21.8)	180 (19.1)	0.065	354 (16.0)	88 (10.8)	**<0.001**
History of TIA	428 (8.2)	60 (6.4)	0.053	169 (7.6)	58 (7.1)	0.622
History of MI	394 (7.6)	51 (5.4)	**0.019**	98 (4.4)	34 (4.2)	0.753
*Comorbid conditions*						
Number of comorbid conditions	2.9 ± 1.6	2.9 ± 1.7	0.329 *	2.7 ± 1.6	2.6 ± 1.5	0.717 *
Arterial hypertension	4424 (85.0)	794 (84.3)	0.569	1701 (76.8)	632 (77.4)	0.745
Chronic heart failure	463 (8.9)	100 (10.6)	0.092	139 (6.3)	37 (4.5)	0.068
Atrial fibrillation	621 (11.9)	115 (12.2)	0.811	291 (13.1)	97 (11.9)	0.355
Diabetes mellitus	2350 (45.2)	389 (41.3)	**0.028**	840 (37.9)	287 (35.1)	0.158
Hypercholesterolemia	2034 (39.1)	327 (34.7)	**0.011**	886 (40.0)	330 (40.4)	0.845
Dementia	528 (10.1)	116 (12.3)	**0.046**	132 (6.0)	39 (4.8)	0.209
Depression	617 (11.9)	128 (13.6)	0.134	257 (11.6)	80 (9.8)	0.159
Anxiety disorders	1601 (30.8)	275 (29.2)	0.335	679 (30.7)	261 (31.9)	0.495
Parkinson’s disease	278 (5.3)	48 (5.1)	0.756	85 (3.8)	33 (4.0)	0.799
Epilepsy	150 (2.9)	29 (3.1)	0.742	51 (2.3)	16 (2.0)	0.567
Bronchial asthma/COPD	1166 (22.4)	231 (24.5)	0.154	503 (22.7)	206 (25.2)	0.148
*Antiplatelet agent related characteristics*						
Initial antiplatelet agent						
Aspirin	3397 (65.3)	706 (74.9)	**<0.001**	1614 (72.9)	674 (82.5)	**<0.001**
Clopidogrel	980 (18.8)	141 (15.0)		350 (15.8)	91 (11.1)	
Ticlopidine	399 (7.7)	63 (6.7)		141 (6.4)	36 (4.4)	
Aspirin + Clopidogrel	428 (8.2)	32 (3.4)		110 (5.0)	16 (2.0)	
New antiplatelet agent user ^b^	621 (11.9)	116 (12.3)	0.740	452 (20.4)	125 (15.3)	**0.001**
Patient’s co-payment (EUR) ^c^	1.5 ± 1.3	1.2 ± 1.0	<0.001 *	1.3 ± 1.1	1.1 ± 1.0	<0.001 *
General practitioner as index prescriber	3950 (75.9)	676 (71.8)	**0.007**	1505 (67.9)	547 (67.0)	0.604
*Cardiovascular co-medication*						
Number of medications	8.3 ± 2.5	8.2 ± 2.5	0.059 *	7.7 ± 2.8	7.8 ± 2.7	0.812 *
Number of CV medications	5.1 ± 2.3	5.0 ± 2.3	0.032 *	4.8 ± 2.3	4.7 ± 2.2	0.363 *
Anticoagulants	1087 (20.9)	213 (22.6)	0.233	471 (21.3)	146 (17.9)	**0.039**
Cardiac glycosides	476 (9.1)	102 (10.8)	0.104	138 (6.2)	28 (3.4)	**0.003**
Antiarrhythmic agents	355 (6.8)	61 (6.5)	0.697	186 (8.4)	52 (6.4)	0.065
Beta-blockers	1079 (20.7)	162 (17.2)	**0.013**	390 (17.6)	158 (19.3)	0.272
Thiazide diuretics	1139 (21.9)	174 (18.5)	**0.019**	484 (21.9)	194 (23.7)	0.267
Loop diuretics	1366 (26.2)	289 (30.7)	**0.005**	395 (17.8)	127 (15.5)	0.139
Mineralocorticoid receptor antagonists	478 (9.2)	97 (10.3)	0.281	112 (5.1)	35 (4.3)	0.380
Calcium channel blockers	1644 (31.6)	274 (29.1)	0.127	680 (30.7)	258 (31.6)	0.642
RAAS inhibitors	4402 (84.6)	786 (83.4)	0.371	1816 (82.0)	655 (80.2)	0.253
Statins	3624 (69.6)	544 (57.7)	**<0.001**	1583 (71.5)	568 (69.5)	0.295
Lipid-lowering agents other than statins ^d^	515 (9.9)	81 (8.6)	0.216	219 (9.9)	87 (10.6)	0.537

In the case of categorical variables, values represent the frequency, and the percentages are provided in parentheses (% of *n*). In the case of continuous variables, means ± standard deviations are provided. TIA—transient ischemic attack; MI–myocardial infarction; COPD—chronic obstructive pulmonary disease; CV—cardiovascular; RAAS—renin-angiotensin-aldosterone system; *p*—statistical significance between adherent and non-adherent patients according to the χ^2^-test; * statistical significance according to the Mann–Whitney U test; in the case of statistical significance (*p* < 0.05), the values are expressed in bold. ^a^ The time period covered by “history”—5 years before the index date of this study. ^b^ New antiplatelet agent user–patient in whom antiplatelet treatment was initiated in association with the diagnosis of peripheral arterial disease. ^c^ Co-payment–calculated as the cost of antiplatelet treatment paid by the patient per month. ^d^ Lipid-lowering agents other than statins–ezetimibe and fibrates.

**Table 2:** Adherence status of non-persistent patients depending on the 12-month period when treatment was discontinued (*n* = 3032).

	0–12 Months *n* = 1436	13–24 Months *n* = 636	25–36 Months *n* = 488	37–48 Months *n* = 335	49–60 Months *n* = 137	*p*
Adherent (PDC ≥ 80%)	1194 (83.1)	355 (55.8)	315 (64.5)	251 (74.9)	100 (73.0)	**<0.001 ^a^**
Non-adherent (PDC < 80%)	242 (16.9)	281 (44.2)	173 (35.5)	84 (25.1)	37 (27.0)	
PDC	91.9 ± 14.8	80.7 ± 16.6	83.8 ± 15.1	86.4 ± 13.5	87.1 ± 13.1	**<0.001 ^b^**

In the case of adherent and non-adherent patients, values represent frequencies (% of n). PDC—proportion of days covered expressed as means ± standard deviations (SD). *p*—statistical significance, ^a^ according to the χ^2^-test, ^b^ according to the Kruskal–Wallis test; in the case of statistical significance (*p* < 0.05), the values are expressed in bold.

**Table 3:** Multivariate analysis of the influence of patient- and medication-associated characteristics on the likelihood of non-adherence (*n* = 9178).

Factor	Persistent (*n* = 6146)	Non-Persistent (*n* = 3032)
*Socio-demographic characteristics*		
Age	1.01 (1.00−1.02)	1.00 (0.98−1.01)
Female sex	0.86 (0.74−1.00)	1.18 (0.98−1.43)
University education	**0.67 (0.47−0.94)**	0.93 (0.67−1.29)
Employed patients	0.96 (0.66−1.40)	0.84 (0.59−1.21)
*History of cardiovascular events* ^a^		
History of ischemic stroke	0.88 (0.73−1.07)	**0.67 (0.51−0.87)**
History of TIA	0.81 (0.61−1.09)	0.99 (0.72−1.38)
History of MI	0.80 (0.58−1.10)	1.06 (0.70−1.61)
*Comorbid conditions*		
Number of comorbid conditions	0.95 (0.79−1.15)	0.97 (0.76−1.22)
Arterial hypertension	1.00 (0.73−1.36)	0.95 (0.68−1.34)
Chronic heart failure	1.19 (0.86−1.64)	0.89 (0.55−1.43)
Atrial fibrillation	1.00 (0.73−1.37)	1.26 (0.87−1.82)
Diabetes mellitus	0.96 (0.75−1.23)	0.90 (0.67−1.21)
Hypercholesterolemia	1.04 (0.81−1.34)	0.99 (0.73−1.33)
Dementia	1.20 (0.89−1.62)	0.88 (0.56−1.39)
Depression	1.29 (0.96−1.72)	0.84 (0.58−1.21)
Anxiety disorders	0.96 (0.75−1.24)	1.07 (0.79−1.45)
Parkinson’s disease	0.93 (0.63−1.36)	1.26 (0.76−2.06)
Epilepsy	1.23 (0.78−1.96)	1.03 (0.54−1.95)
Bronchial asthma/COPD	1.20 (0.92−1.56)	1.26 (0.92−1.72)
*Antiplatelet agent related characteristics*		
Initial antiplatelet agent		
Aspirin	1.00	1.00
Clopidogrel	0.90 (0.71−1.15)	**0.71 (0.53−0.97)**
Ticlopidine	1.17 (0.80−1.69)	0.68 (0.43−1.09)
Aspirin + Clopidogrel	**0.52 (0.34−0.78)**	**0.47 (0.26−0.84)**
New antiplatelet agent user ^b^	0.94 (0.72−1.23)	**0.69 (0.53−0.90)**
Patient’s co-payment (EUR) ^c^	**0.83 (0.75−0.91)**	0.93 (0.83−1.05)
General practitioner as index prescriber	**0.79 (0.67−0.94)**	**0.81 (0.67−0.98)**
*Cardiovascular co-medication*		
Number of medications	1.00 (0.96−1.04)	1.04 (0.99−1.09)
Number of CV medications	1.00 (0.93−1.07)	1.00 (0.92−1.09)
Anticoagulants	1.18 (0.97−1.44)	0.83 (0.65−1.05)
Cardiac glycosides	0.97 (0.74−1.27)	**0.58 (0.37−0.91)**
Antiarrhythmic agents	0.97 (0.70−1.34)	0.74 (0.51−1.07)
Beta-blockers	0.84 (0.68−1.04)	1.11 (0.87−1.42)
Thiazide diuretics	0.87 (0.71−1.06)	1.05 (0.84−1.31)
Loop diuretics	1.22 (0.99−1.50)	0.87 (0.66−1.14)
Mineralocorticoid receptor antagonists	1.02 (0.77−1.35)	1.17 (0.75−1.82)
Calcium channel blockers	0.95 (0.79−1.15)	0.95 (0.77−1.18)
RAAS inhibitors	1.12 (0.89−1.41)	0.79 (0.61−1.02)
Statins	**0.67 (0.56−0.80)**	0.95 (0.78−1.16)
Lipid-lowering agents other than statins ^d^	0.99 (0.76−1.30)	1.06 (0.79−1.42)

Values represent odds ratios (95% confidence intervals). In the case of statistical significance (*p* < 0.05), the values are expressed in bold. TIA–transient ischemic attack; MI–myocardial infarction; COPD–chronic obstructive pulmonary disease; CV—cardiovascular; RAAS—renin-angiotensin-aldosterone system. ^a^ The time period covered by “history”—5 years before the index date of this study. ^b^ New antiplatelet agent user–patient in whom antiplatelet treatment was initiated in association with the diagnosis of peripheral arterial disease. ^c^ Co-payment–calculated as the cost of antiplatelet treatment paid by the patient per month. ^d^ Lipid-lowering agents other than statins–ezetimibe and fibrates.

## Data Availability

The data that support the findings of this study are available from the General Health Insurance Company but restrictions apply to the availability of these data, which were used under license for the current study, and so are not publicly available. Data are however available from the authors upon reasonable request and with permission of the General Health Insurance Company.

## Supplementary Materials

The following are available online at https://www.mdpi.com/article/10.3390/biomedicines9121800/s1, Supplementary Table S1 Multivariate analysis of the influence of patient- and medication-associated characteristics on the likelihood of non-adherence in the whole study group which included both persistent and non-persistent patients (*n* = 9178); Supplementary Table S2 Multivariate analysis of the influence of patient- and medication-associated characteristics on the likelihood of non-adherence evaluated in models using different thresholds defining non-adherence (*n* = 9178); Supplementary Table S3 Multivariate analysis of the influence of patient- and medication-associated characteristics on the likelihood of non-adherence evaluated in the model with a shorter 3-year follow-up period and with a standard 80% threshold defining non-adherence (*n* = 9178).
