# Supplementary material for: Adherence to Antiplatelet Medications among Persistent and Non-Persistent Older Patients with Peripheral Arterial Disease
Source: Biomedicines. 2021 Nov 30;9(12):1800. doi: 10.3390/biomedicines9121800 (PMC8698509; doi:10.3390/biomedicines9121800)
Supplement: Supplementary file 1 [file biomedicines-09-01800-s001.zip › Supplementary Table S1.pdf]

Supplementary Table S1 Multivariate analysis of the influence of patient- and medication-associated characteristics on the likelihood of non-adherence in the whole study group which included both persistent and non-persistent patients (n = 9178).

**Factor**	**OR (95% CI)**
Socio-demographic characteristics	
Age	1.0 (0.99–1.01)
Female sex	1.05 (0.94–1.18)
University education	0.81 (0.64–1.01)
Employed patients	0.94 (0.73–1.22)
History of cardiovascular events[a]	
History of ischemic stroke	**0.79 (0.67–0.91)**
History of TIA	0.91 (0.73–1.12)
History of MI	0.87 (0.68–1.11)
Comorbid conditions	
Number of comorbid conditions	0.94 (0.82–1.09)
Arterial hypertension	0.96 (0.77–1.20)
Chronic heart failure	1.08 (0.83–1.40)
Atrial fibrillation	1.17 (0.93–1.49)
Diabetes mellitus	0.91 (0.76–1.10)
Hypercholesterolemia	1.05 (0.87–1.27)
Dementia	1.07 (0.84–1.37)
Depression	1.10 (0.88–1.38)
Anxiety disorders	1.05 (0.86–1.27)
Parkinson's disease	1.03 (0.77–1.39)
Epilepsy	1.15 (0.80–1.67)
Bronchial asthma/COPD	**1.27 (1.04–1.55)**
Antiplatelet agent related characteristics	
Initial antiplatelet agent	
Aspirin	1.00
Clopidogrel	**0.79 (0.66–0.95)**
Ticlopidine	0.94 (0.70–1.25)
Aspirin + Clopidogrel	**0.46 (0.33–0.64)**
New antiplatelet agent user[b]	0.86 (0.71–1.03)
Patient´s co-payment (EUR)[c]	**0.86 (0.80–0.93)**
General practitioner as index prescriber	**0.78 (0.69–0.88)**

(Table continued)

Factor	OR (95% CI)
Cardiovascular co-medication	
Number of medications	1.01 (0.98–1.04)
Number of CV medications	0.99 (0.93–1.04)
Anticoagulants	1.05 (0.90–1.22)
Cardiac glycosides	0.86 (0.69–1.08)
Antiarrhythmic agents	0.91 (0.71–1.16)
Beta-blockers	0.94 (0.81–1.10)
Thiazide diuretics	0.97 (0.84–1.12)
Loop diuretics	1.07 (0.91–1.26)
Mineralocorticoid receptor antagonists	1.06 (0.84–1.33)
Calcium channel blockers	0.98 (0.85–1.12)
RAAS inhibitors	0.97 (0.82–1.14)
Statins	**0.79 (0.69–0.89)**
Lipid lowering agents other than statins[d]	1.04 (0.86–1.26)

Values represent odds ratios (95% confidence intervals). In the case of statistical significance ($p<0.05$), the values are expressed in bold. TIA – transient ischemic attack; MI – myocardial infarction; COPD – chronic obstructive pulmonary disease; CV – cardiovascular; RAAS – renin angiotensin aldosterone system. [a]The time period covered by "history" – 5 years before the index date of this study. [b]New antiplatelet agent user – patient in whom antiplatelet treatment was initiated in association with the diagnosis of peripheral arterial disease. [c]Co-payment – calculated as the cost of antiplatelet treatment paid by the patient per month. [d]Lipid lowering agents other than statins – ezetimibe and fibrates.
