# Supplementary material for: Adherence to Antiplatelet Medications among Persistent and Non-Persistent Older Patients with Peripheral Arterial Disease
Source: Biomedicines. 2021 Nov 30;9(12):1800. doi: 10.3390/biomedicines9121800 (PMC8698509; doi:10.3390/biomedicines9121800)
Supplement: Supplementary file 1 [file biomedicines-09-01800-s001.zip › Supplementary Table S2.pdf]

Supplementary Table S2 Multivariate analysis of the influence of patient- and medication-associated characteristics on the likelihood of non-adherence evaluated in models using different thresholds defining non-adherence (n = 9178)

Factor	PDC threshold defining non-adherence							
	PDC<50%		PDC<60%		PDC<70%		PDC<90%	
	Persistent n=6146	Non-persistent n=3032	Persistent n=6146	Non-persistent n=3032	Persistent n=6146	Non-persistent n=3032	Persistent n=6146	Non-persistent n=3032
Socio-demographic characteristics								
Age	1.02 (0.97–1.06)	1.02 (0.97–1.07)	1.00 (0.98–1.03)	1.00 (0.97–1.02)	1.00 (0.99–1.02)	1.00 (0.98–1.02)	1.00 (1.00–1.01)	1.01 (0.99–1.02)
Female sex	0.87 (0.46–1.63)	1.08 (0.58–2.01)	1.24 (0.92–1.68)	1.25 (0.91–1.71)	1.01 (0.82–1.24)	1.22 (0.97–1.53)	0.90 (0.80–1.01)	1.14 (0.96–1.35)
University education	0.83 (0.19–3.60)	0.99 (0.33–2.91)	0.57 (0.26–1.25)	1.09 (0.64–1.88)	**0.60 (0.37–0.99)**	0.73 (0.48–1.11)	0.83 (0.65–1.05)	1.08 (0.81–1.44)
Employed patients	0.59 (0.08–4.42)	0.50 (0.12–2.15)	0.61 (0.25–1.52)	0.50 (0.24–1.04)	0.85 (0.50–1.44)	0.90 (0.58–1.38)	0.96 (0.72–1.27)	0.95 (0.69–1.31)
History of cardiovascular events[a]								
History of ischemic stroke	0.95 (0.42–2.12)	0.80 (0.35–1.83)	0.89 (0.60–1.30)	0.70 (0.45–1.11)	0.89 (0.69–1.16)	**0.71 (0.52–0.98)**	**0.86 (0.74–0.99)**	**0.76 (0.60–0.95)**
History of TIA	0,82 (0.61–1.03)	0.58 (0.17–1.95)	**0.44 (0.20–0.95)**	0.84 (0.48–1.48)	0.73 (0.48–1.11)	1.15 (0.79–1.68)	0.85 (0.68–1.06)	1.01 (0.76–1.36)
History of MI	1.10 (0.32–3.76)	1.02 (0.23–4.47)	1.06 (0.58–1.91)	0.63 (0.27–1.47)	0.91 (0.60–1.39)	1.08 (0.65–1.79)	0.94 (0.75–1.18)	0.96 (0.66–1.40)
Comorbid conditions								
Number of comorbid conditions	0.55 (0.21–1.42)	0.78 (0.34–1.76)	0.85 (0.58–1.26)	0.91 (0.61–1.36)	0.91 (0.70–1.18)	1.11 (0.84–1.45)	1.01 (0.87–1.16)	0.92 (0.75–1.14)
Arterial hypertension	3.93 (0.81–19.13)	0.47 (0.16–1.41)	1.43 (0.76–2.68)	0.81 (0.46–1.41)	1.19 (0.78–1.80)	0.82 (0.55–1.22)	0.88 (0.69–1.11)	1.18 (0.87–1.60)
Chronic heart failure	1.39 (0.31–6.22)	2.22 (0.54–9.16)	0.90 (0.45–1.78)	1.22 (0.57–2.64)	1.18 (0.77–1.83)	0.93 (0.53–1.63)	1.23 (0.96–1.59)	1.12 (0.75–1.69)
Atrial fibrillation	4.09 (0.98–14.81)	1.34 (0.36–5.02)	2.20 (0.92–4.29)	1.40 (0.76–2.59)	1.41 (0.94–2.13)	0.92 (0.59–1.44)	0.95 (0.75–1.22)	1.17 (0.84–1.63)
Diabetes mellitus	1.34 (0.42–4.30)	1.71 (0.64–4.61)	1.16 (0.71–1.89)	1.19 (0.73–1.95)	1.07 (0.77–1.48)	0.91 (0.64–1.28)	0.90 (0.74–1.08)	0.97 (0.75–1.26)
Hypercholesterolemia	1.91 (0.57–6.33)	1.25 (0.45–3.43)	1.30 (0.78–2.17)	1.00 (0.60–1.66)	1.08 (0.76–1.52)	0.84 (0.59–1.19)	1.00 (0.82–1.22)	1.06 (0.81–1.39)
Dementia	2.16 (0.58–8.00)	1.33 (0.30–5.85)	0.98 (0.52–1.84)	0.79 (0.35–1.80)	1.23 (0.82–1.83)	0.58 (0.32–1.04)	1.04 (0.82–1.32)	0.89 (0.60–1.33)
Depression	0.57 (0.10–3.23)	0.79 (0.21–2.94)	0.87 (0.46–1.64)	1.25 (0.69–2.26)	0.92 (0.61–1.40)	0.90 (0.59–1.37)	1.18 (0.94–1.48)	0.86 (0.62–1.20)

(Table continued)

Factor	PDC threshold defining non-adherence							
	PDC<50%		PDC<60%		PDC<70%		PDC<90%	
	Persistent n=6146	Non-persistent n=3032	Persistent n=6146	Non-persistent n=3032	Persistent n=6146	Non-persistent n=3032	Persistent n=6146	Non-persistent n=3032
Anxiety disorders	2.08 (0.65–6.69)	1.84 (0.67–5.04)	0.96 (0.57–1.60)	1.24 (0.75–2.05)	0.94 (0.67–1.33)	0.97 (0.68–1.37)	1.02 (0.84–1.24)	1.07 (0.81–1.40)
Parkinson's disease	2.42 (0.49–11.86)	2.49 (0.61–10.07)	1.18 (0.54–2.58)	0.67 (0.26–1.75)	1.19 (0.72–1.97)	0.59 (0.31–1.16)	0.92 (0.68–1.24)	1.18 (0.75–1.86)
Epilepsy	1.82 (0.19–17.86)	**4.88 (1.17–20.26)**	1.30 (0.47–3.59)	**2.64 (1.11–6.26)**	1.19 (0.62–2.28)	1.28 (0.62–2.63)	0.94 (0.64–1.36)	0.81 (0.45–1.44)
Bronchial asthma/COPD	1.12 (0.31–4.04)	1.90 (0.67–5.42)	1.21 (0.71–2.05)	1.38 (0.82–2.34)	1.01 (0.71–1.46)	1.14 (0.79–1.65)	1.14 (0.93–1.40)	**1.39 (1.04–1.85)**
Antiplatelet agent related characteristics								
Initial antiplatelet agent								
Aspirin	1.00	1.00	1.00	1.00	1.00	1.00	1.00	1.00
Clopidogrel	0.60 (0.17–2.09)	0.50 (0.19–1.32)	0.98 (0.61–1.57)	0.77 (0.46–1.29)	0.83 (0.59–1.16)	0.72 (0.49–1.04)	**0.76 (0.63–0.91)**	**0.71 (0.55–0.92)**
Ticlopidine	7.87 (0.92–34.98)	0.29 (0.06–1.41)	1.44 (0.69–2.99)	0.85 (0.38–1.92)	1.41 (0.85–2.35)	0.97 (0.55–1.71)	0.90 (0.68–1.20)	0.67 (0.41–1.02)
Aspirin + Clopidogrel	1.00 (0.22–4.55)	0.77 (0.52–1.21)	0.43 (0.17–1.09)	0.14 (0.02–1.02)	**0.40 (0.21–0.75)**	**0.24 (0.08–0.66)**	**0.42 (0.32–0.57)**	**0.48 (0.30–0.76)**
New antiplatelet agent user[b]	0.88 (0.30–2.54)	0.84 (0.36–1.98)	1.01 (0.61–1.67)	0.71 (0.45–1.12)	0.90 (0.63–1.28)	0.83 (0.61–1.14)	0.90 (0.73–1.10)	**0.69 (0.54–0.87)**
Patient´s co-payment (EUR)[c]	**0.50 (0.29–0.85)**	1.32 (0.98–1.76)	**0.79 (0.64–0.98)**	0.89 (0.72–1.11)	**0.78 (0.68–0.91)**	**0.86 (0.73–0.99)**	**0.86 (0.80–0.92)**	0.96 (0.88–1.06)
General practitioner as index prescriber	0.53 (0.27–1.01)	0.72 (0.39–1.30)	**0.70 (0.51–0.96)**	**0.72 (0.53–0.98)**	**0.70 (0.56–0.88)**	0.81 (0.65–1.02)	**0.76 (0.67–0.87)**	0.97 (0.82–1.15)
Cardiovascular co-medication								
Number of medications	0.93 (0.78–1.09)	1.14 (0.98–1.34)	1.01 (0.93–1.09)	1.07 (0.99–1.15)	0.98 (0.93–1.04)	1.03 (0.97–1.09)	0.99 (0.96–1.02)	**1.05 (1.00–1.09)**
Number of CV medications	1.17 (0.88–1.55)	0.92 (0.70–1.22)	0.99 (0.86–1.13)	0.97 (0.84–1.12)	0.98 (0.89–1.08)	1.05 (0.95–1.16)	1.00 (0.94–1.05)	0.97 (0.90–1.05)
Anticoagulants	1.22 (0.55–2.67)	0.48 (0.19–1.22)	1.22 (0.84–1.79)	0.70 (0.46–1.06)	1.22 (0.94–1.59)	0.80 (0.60–1.06)	1.15 (0.98–1.34)	0.81 (0.64–1.01)
Cardiac glycosides	0.88 (0.31–2.45)	0.85 (0.18–4.16)	1.12 (0.68–1.86)	0.77 (0.36–1.62)	0.91 (0.64–1.30)	0.66 (0.38–1.16)	0.84 (0.68–1.04)	**0.67 (0.46–0.98)**
Antiarrhythmic agents	0.59 (0.16–2.21)	0.56 (0.12–2.71)	0.48 (0.18–1.02)	0.91 (0.50–1.68)	0.63 (0.39–1.02)	0.80 (0.51–1.25)	0.92 (0.71–1.18)	1.11 (0.80–1.53)

(Table continued)

Factor	PDC threshold defining non-adherence							
	PDC<50%		PDC<60%		PDC<70%		PDC<90%	
	Persistent n=6146	Non-persistent n=3032	Persistent n=6146	Non-persistent n=3032	Persistent n=6146	Non-persistent n=3032	Persistent n=6146	Non-persistent n=3032
Beta-blockers	0.31 (0.04–1.03)	1.33 (0,62–2.86)	0.81 (0.53–1.24)	1.39 (0.94–2.05)	0.94 (0.71–1.25)	1.07 (0.80–1.42)	0.92 (0.78–1.08)	0.95 (0.76–1.19)
Thiazide diuretics	1.63 (0.77–3.43)	1.82 (0.94–3.53)	0.93 (0.63–1.38)	1.03 (0.71–1.49)	0.81 (0.61–1.06)	0.91 (0.70–1.19)	1.02 (0.87–1.18)	0.92 (0.75–1.13)
Loop diuretics	0.90 (0.37–2.15)	0.58 (0.22–1.54)	0.92 (0.61–1.39)	0.83 (0.52–1.31)	1.23 (0.93–1.62)	0.73 (0.50–1.05)	1.21 (0.98–1.47)	0.83 (0.65–1.06)
Mineralocorticoid receptor antagonists	0.70 (0.20–2.39)	0.46 (0.06–3.74)	0.80 (0.44–1.45)	1.17 (0.56–2.44)	1.06 (0.73–1.54)	0.87 (0.49–1.54)	1.09 (0.87–1.35)	1.16 (0.79–1.72)
Calcium channel blockers	0.52 (0.22–1.23)	1.22 (0.62–2.40)	0.87 (0.60–1.26)	0.92 (0.65–1.32)	0.84 (0.65–1.08)	0.83 (0.64–1.07)	0.95 (0.82–1.10)	1.04 (0.85–1.26)
RAAS inhibitors	1.16 (0.43–3.11)	0.69 (0.31–1.54)	1.44 (0.89–2.32)	0.77 (0.50–1.17)	1.35 (0.98–1.86)	0.74 (0.51–1.03)	1.12 (0.93–1.34)	0.79 (0.62–1.00)
Statins	0.57 (0.28–1.15)	0.73 (0.39–1.34)	**0.54 (0.39–0.76)**	0.87 (0.63–1.21)	**0.62 (0.49–0.78)**	0.89 (0.71–1.13)	**0.78 (0.68–0.90)**	1.00 (0.84–1.20)
Lipid lowering agents other than statins[d]	0.77 (0.22–2.74)	1.75 (0.79–3.87)	1.33 (0.82–2.16)	0.97 (0.60–1.57)	0.99 (0.68–1.42)	1.04 (0.74–1.46)	0.94 (0.77–1.15)	1.00 (0.77–1.30)

Values represent odds ratios (95% confidence intervals). In the case of statistical significance ($p<0.05$), the values are expressed in bold. PDC – Proportion of Days Covered; TIA – transient ischemic attack; MI – myocardial infarction; COPD – chronic obstructive pulmonary disease; CV – cardiovascular; RAAS – renin angiotensin aldosterone system. [a]The time period covered by "history" – 5 years before the index date of this study. [b]New antiplatelet agent user – patient in whom antiplatelet treatment was initiated in association with the diagnosis of peripheral arterial disease. [c]Co-payment – calculated as the cost of antiplatelet treatment paid by the patient per month. [d]Lipid lowering agents other than statins – ezetimibe and fibrates.
