# Supplementary material for: Adherence to Antiplatelet Medications among Persistent and Non-Persistent Older Patients with Peripheral Arterial Disease
Source: Biomedicines. 2021 Nov 30;9(12):1800. doi: 10.3390/biomedicines9121800 (PMC8698509; doi:10.3390/biomedicines9121800)
Supplement: Supplementary file 1 [file biomedicines-09-01800-s001.zip › Supplementary Table S3.pdf]

**Supplementary Table S3 Multivariate analysis of the influence of patient- and medication-associated characteristics on the likelihood of non-adherence evaluated in the model with a shorter 3-year follow-up period and with a standard 80% threshold defining non-adherence (n = 9178).**

| **Factor** | **Persistent n = 6419** | **Non-persistent n = 2759** |
|---|---|---|
| *Socio-demographic characteristics* | | |
| Age | 1.01 (0.99–1.02) | 1.00 (0.98–1.01) |
| Female sex | 0.87 (0.75–1.01) | 1.16 (0.96–1.39) |
| University education | 0.77 (0.56–1.06) | 0.98 (0.71–1.35) |
| Employed patients | 0.97 (0.67–1.38) | 0.89 (0.62–1.27) |
| *History of cardiovascular events*[a] | | |
| History of ischemic stroke | 0.89 (0.74–1.07) | **0.73 (0.56–0.94)** |
| History of TIA | 0.86 (0.65–1.13) | 0.97 (0.70–1.35) |
| History of MI | 0.84 (0.62–1.14) | 1.12 (0.74–1.68) |
| *Comorbid conditions* | | |
| Number of comorbid conditions | 0.95 (0.79–1.14) | 1.00 (0.80–1.26) |
| Arterial hypertension | 1.03 (0.76–1.39) | 0.97 (0.70–1.35) |
| Chronic heart failure | 1.21 (0.88–1.66) | 0.80 (0.50–1.29) |
| Atrial fibrillation | 0.98 (0.72–1.33) | 1.26 (0.87–1.82) |
| Diabetes mellitus | 0.97 (0.76–1.22) | 0.87 (0.65–1.16) |
| Hypercholesterolemia | 0.97 (0.76–1.25) | 0.95 (0.70–1.27) |
| Dementia | 1.11 (0.83–1.49) | 0.84 (0.53–1.32) |
| Depression | 1.26 (0.95–1.67) | 0.79 (0.55–1.14) |
| Anxiety disorders | 1.01 (0.79–1.29) | 1.01 (0.75–1.35) |
| Parkinson’s disease | 1.14 (0.79–1.63) | 1.16 (0.71–1.91) |
| Epilepsy | 1.16 (0.73–1.83) | 0.95 (0.50–1.80) |
| Bronchial asthma/COPD | 1.23 (0.95–1.59) | 1.26 (0.92–1.72) |
| *Antiplatelet agent related characteristics* | | |
| Initial antiplatelet agent | | |
| Aspirin | 1.00 | 1.00 |
| Clopidogrel | 0.79 (0.62–1.01) | **0.71 (0.53–0.96)** |
| Ticlopidine | 1.27 (0.88–1.83) | 0.65 (0.40–1.03) |
| Aspirin + Clopidogrel | **0.43 (0.28–0.65)** | **0.44 (0.25–0.79)** |
| New antiplatelet agent user[b] | 0.96 (0.74–1.25) | **0.71 (0.54–0.92)** |
| Patient´s co-payment (EUR)[c] | **0.81 (0.74–0.90)** | 0.93 (0.83–1.04) |
| General practitioner as index prescriber | **0.77 (0.65–0.91)** | 0.85 (0.70–1.02) |

*(Table continued)*

| Factor | Persistent n = 6419 | Non-persistent n = 2759 |
|---|---|---|
| *Cardiovascular co-medication* | | |
| Number of medications | 0.99 (0.95–1.03) | 1.03 (0.98–1.08) |
| Number of CV medications | 1.01 (0.94–1.08) | 0.99 (0.91–1.08) |
| Anticoagulants | 1.15 (0.95–1.40) | 0.81 (0.64–1.03) |
| Cardiac glycosides | 0.92 (0.70–1.19) | 0.67 (0.43–1.04) |
| Antiarrhythmic agents | 1.10 (0.81–1.50) | 0.74 (0.51–1.07) |
| Beta-blockers | 0.84 (0.68–1.03) | 1.14 (0.90–1.45) |
| Thiazide diuretics | 0.83 (0.68–1.01) | 1.03 (0.83–1.29) |
| Loop diuretics | 1.16 (0.95–1.42) | 0.94 (0.72–1.23) |
| Mineralocorticoid receptor antagonists | 0.98 (0.75–1.30) | 0.99 (0.63–1.55) |
| Calcium channel blockers | 0.95 (0.79–1.14) | 0.96 (0.78–1.19) |
| RAAS inhibitors | 1.15 (0.91–1.44) | 0.82 (0.63–1.06) |
| Statins | **0.72 (0.61–0.85)** | 0.94 (0.77–1.14) |
| Lipid lowering agents other than statins[d] | 1.04 (0.81–1.35) | 1.14 (0.85–1.51) |

Values represent odds ratios (95% confidence intervals). In the case of statistical significance ($p<0.05$), the values are expressed in bold. TIA – transient ischemic attack; MI – myocardial infarction; COPD – chronic obstructive pulmonary disease; CV – cardiovascular; RAAS – renin angiotensin aldosterone system. [a]The time period covered by "history" – 5 years before the index date of this study. [b]New antiplatelet agent user – patient in whom antiplatelet treatment was initiated in association with the diagnosis of peripheral arterial disease. [c]Co-payment – calculated as the cost of antiplatelet treatment paid by the patient per month. [d]Lipid lowering agents other than statins – ezetimibe and fibrates.
